# Adherence to environmental control recommendation among children with asthma and allergic rhinitis

**DOI:** 10.1186/1939-4551-8-S1-A118

**Published:** 2015-04-08

**Authors:** Ana Paula Willy-Fabro, Tatiane Pavan-Ramos, Ana Carolina Rozalem, Marcia Carvalho Mallozi, Dirceu Solé, Gustavo Falbo Wandalsen

**Affiliations:** 1 Federal University of Sao Paulo, Brazil

## Background

Allergen exposure in sensitized patients has been associated to asthma and allergic rhinitis exacerbations, to severe symptoms and to medication need. Indoor allergens control is important in the management of atopic diseases in children, as they spend a large proportion of their time at home.

## Methods

A cross-sectional study design was adopted in order to access the adherence to environmental control recommendation in the management of asthma and allergic rhinitis. We selected patients aged between 5 and 18 years, who had the diagnosis of allergic asthma and/or allergic rhinitis and who were in regular follow-up for more than 6 months.

## Results

96 patients were recruited. The median age was 10.3 years, and 66% (64) were male. All patients had a diagnosis of allergic rhinitis and 84 patients (87.5%) had a diagnosis of asthma. The responder was the child’s mother in 87% of cases. 56% of responders had secondary or tertiary education and 45% of them were atopic. 76% of responders recognized that their children’s doctors had instructed them about environmental control measures. In order to reduce house dust, 91% of houses had hard flooring and 97% were cleaned by moping the floor daily (52%) or weekly (45%). Moreover, 92% of housekeepers used to wash bedding every week, 72% minimized dust-accumulating objects, 90% removed soft toys, 83% removed rugs and 51% removed curtains. However, only 33% encased mattresses and pillows in impermeable covers. Other practices included ventilating the house by opening the windows every day in 96%, carefully cleaning the house to avoid cockroaches in 95% and chemical controlling cockroaches in 26% of cases. For pet allergen avoidance, 54% of families chose not to have pets. Among the families who had them, 52% kept the pet out of the main living areas and 27% used to wash the pet every week. Finally, 33% of families had at least one member who smoked, but only 3% of caregivers quit smoking. Among non-smoking caregivers, 29% reduced their child’s exposure to sources of passive smoke outside the family.

## Conclusions

Caregivers understood and applied general allergen-avoidance measures in their homes. Despite of that, the majority of caregivers did not implement some specific measures with proven benefit in reducing allergen levels, such as encasing mattresses and pillows in impermeable covers, chemical controlling cockroaches and quitting tobacco smoke.

